# Dataset of greenhouse gas fluxes and soil properties from a biochar application frequency and dosage experiment in an upland agroecosystem in Guizhou, China (2020-2023)

**DOI:** 10.1016/j.dib.2026.112754

**Published:** 2026-04-12

**Authors:** Lihong Song, Yan Zhang

**Affiliations:** a College of New Energy Materials and Chemistry, Leshan Normal University, Leshan 614000, China; b College of Agriculture, Guizhou University, Guiyang 550025, China

**Keywords:** Biochar, Application frequency, Greenhouse gas fluxes, Upland soil, Soil microbial biomass carbon and nitrogen, Medium-term monitoring dataset

## Abstract

This data article describes a dataset from a field experiment investigating the effects of biochar on an upland agroecosystem in Guizhou Province, China. An in-situ field experiment was established in 2020 with different biochar application methods (single versus continuous annual application) at five rates (0, 5, 10, 20, 50 t ha^−1^). The data were collected during a 1.5-year monitoring period from April 2022 to October 2023. The dataset includes soil greenhouse gas fluxes (CO_2_, CH_4_, and N_2_O) measured using the static chamber–gas chromatography method. It also provides a profile of soil biophysiochemical properties, including soil organic carbon, bulk density, water content, pH, electrical conductivity, soil available nutrients (available nitrogen, phosphorus, and potassium), soil microbial biomass carbon and nitrogen, and the activities of four enzymes (phosphatase, sucrase, catalase, urease). This dataset captures the interactions between soil biogeochemistry and management practices over an extended period. It serves as a useful resource for biogeochemical model calibration, meta-analyses, and the development of sustainable biochar management strategies aimed at climate change mitigation in agricultural soils.

Specifications Table

**Table:** 

Subject	Earth & Environmental Sciences
Specific subject area	Soil Science; Agroecology; Climate Change Mitigation
Type of data	Table (CSV file)
Data collection	Data were collected from a field experiment established in 2020 in an upland agroecosystem in Guizhou Province, China. Soil and gas measurements were conducted from April 2022 to October 2023. Soil greenhouse gas (GHG) fluxes (CO_2_, CH_4_, and N_2_O) were measured using the static chamber method. Gas sampling began in April 2022 following the final biochar application and was conducted approximately 10-day intervals. At each sampling event, gas samples were collected at 0, 10, 20, and 30 min after chamber closure using 60 mL polypropylene syringes. Gas concentrations were determined using a gas chromatograph (GC-2014, Shimadzu, Japan). Soil samples (0–10 cm) were collected adjacent to each chamber. Soil sampling was conducted in July and October 2022, and in January, April, July, and October 2023. Samples were transported to the laboratory and processed within 24 h. All measurements were conducted following consistent protocols across treatments.
Data source location	Experimental Farm of Guizhou University, Guizhou Province, China (106°39′32″ E, 26°26′20″ N)
Data accessibility	Repository name: Figshare Direct URL to data: https://figshare.com/s/1e0bd63da8e91ec728a7
Related research article	Y. Zhang, L. Song, K. Li, Y. Liu, T. Long, T. Zhang, Z. Chen, S. Yang. Continuous biochar application results in higher greenhouse gas emissions than a single biochar application in an upland agroecosystem. Journal of Environmental Sciences, 161 (2026) 485–493. https://doi.org/10.1016/j.jes.2025.05.055

## 1 Value of the Data

• The dataset provides a continuous 1.5-year record of soil greenhouse gas fluxes under different biochar application strategies. It includes both previously published first-year data and additional six months of unpublished observations.
• The dataset combines gas flux measurements with soil physicochemical and biological properties collected from the same plots.
• The data can be used for model calibration, comparison of biochar application strategies, and analysis of soil carbon and nitrogen dynamics.
• The structure of the dataset allows calculation of cumulative emissions and examination of temporal patterns across treatments.

## 2 Background

Biochar has been widely used as a soil amendment in agricultural systems. The application of biochar is a promising strategy for carbon sequestration and sustainable soil management [1,2]. However, the dynamics of its impact on greenhouse gas (GHG) emissions remain uncertain, particularly as the biochar ages in the soil [3]. Most existing field studies focus on the initial application effects or short-term responses [4]. The frequency of biochar application may lead to different outcomes for soil biogeochemistry over time due to differences in biochar aging, priming effects, and interactions with the soil microbiome [5].

While our related research article [6] analyzed the outcomes from the first year following a three-year application trial, a comprehensive understanding requires long-term monitoring. The processes influenced by biochar, such as soil organic matter stabilization, microbial community adaptation, and nutrient release, unfold over multiple seasons. This dataset records the dynamics of soil GHG fluxes and soil biophysiochemical properties for 1.5 years after the final biochar application. This provides a useful resource for unraveling the effects of different biochar management practices, filling the gap in the existing literature. The 1.5-year post-application period (covering at least one full subsequent growing season) is crucial to capture the legacy effect and the trend of biochar impacts, as key biogeochemical interactions, and the stabilization of applied materials often manifest within this timeframe [3,4].

## 3 Data Description

### 3.1 Data structure and organization

The dataset is available as a single Microsoft Excel workbook (.xlsx) file on Figshare [7]. The dataset contains a total of 675 records and 24 variables. The primary data sheet contains columns for treatment codes, replication, sampling date, GHG fluxes (instantaneous rates and cumulative emissions between sampling points), and soil properties.

### 3.2 Data organization logic and reusability

A key feature of this dataset is the inclusion of both instantaneous flux rates (e.g., CO_2_ rate) and calculated cumulative emissions between consecutive sampling dates (e.g., CO_2_ cumulation). The cumulative values are derived from the instantaneous rates using the integration method over the time interval between two sampling events. This structure is designed to facilitate the calculation of total seasonal or annual cumulative emissions by future users. A detailed description of this calculation is also provided within the Metadata worksheet of the dataset file to ensure clarity and reproducibility.

### 3.3 Uniqueness compared to the related research article

Zhang et al. [6] focused on the scientific analysis and interpretation of data covering the first year after the final biochar application (April 2022 to March 2023). Their conclusions on global warming potential and treatment effects are based on this subset of the data.

The present article provides the raw 1.5-year dataset (April 2022 to October 2023). This includes underlying data for the period analyzed in Zhang et al. [6]. It also includes the previously unreleased and unanalyzed data from the subsequent six months (April to October 2023). This extended data could be used to investigate the inter-annual variability and the decay rate of the biochar effects over a more meaningful timeframe. Crucially, while the related research article [6] provides an in-depth analysis of the underlying mechanisms (e.g., GHG fluxes linked to application frequency), this complete dataset serves as the foundational resource for independently testing, reproducing, or extending those mechanistic interpretations regarding total carbon/nitrogen inputs, and biochar aging stages.

## 4 Experimental Design, Materials and Methods

### 4.1 Site description and experimental design

The field experiment was established at the Experimental Farm of Guizhou University, China (106°39′32″E, 26°26′20″N; 1108 m a.s.l). The area had a mid-subtropical monsoon climate, with mean annual temperature of 14.9 °C, mean annual precipitation of 1200 mm. The soil was classified as yellow soil (Haplic Alisol). The monitoring period of 1.5 years (April 2022 - October 2023) was designed to build upon the first-year data reported in [6]. It aims to capture the ecosystem response throughout an entire additional growing season (April - October 2023), thereby providing a medium-term perspective on the persistence of biochar effects.

A randomized complete block design with three replicates was employed. The experiment included five biochar application rates (0, 5, 10, 20, 50 t ha^−1^) and two application methods: a single application of the total amount in April 2020, and a continuous application where the total amount was split into three equal annual doses applied in April of 2020, 2021, and 2022. Each plot measured 15 m^2^ (5 m × 3 m), with a 0.5 m buffer zone between plots. The cropping system was a maize-rapeseed rotation, and field management followed standard local practices. The biochar was produced from maize straw pyrolyzed at 500 °C for 6 h under anaerobic conditions. It had a carbon content of 657.5 g/kg, a C/N ratio of 62.5, a pH of 7.98, a particle size of 0.3 cm, and an ash content of 29.5 %.

### 4.2 Greenhouse gas flux measurements

Soil GHG fluxes (CO_2_, CH_4_, and N_2_O) were measured using the static chamber-gas chromatography method [6,8]. The static chamber system consisted of a base (0.5 m × 0.5 m × 0.2 m) and a lid (0.5 m × 0.5 m × 0.5 m). One base was permanently installed in each plot. No vegetation was present inside the chamber during sampling period. The top edge of the base had a U‑shaped groove (1 cm wide × 1 cm deep) to seat the lid. The chamber system was insulated to minimize internal temperature fluctuations relative to the outside. A rubber tube connected the lid interior to a three-way valve on the outside. For gas sampling, water was poured into the groove to create an airtight seal. Once the lid was placed on the base, a 60 mL plastic syringe with a three-way valve was attached to the corresponding valve on the lid for immediate sample collection. Gas sampling began in April 2022 following the final biochar application approximately 10-day intervals on clear, windless mornings between 9:00 and 11:00 AM. During each sampling event, four gas samples were collected from the chamber headspace at 10-minute intervals using a 60 mL syringe. Gas concentrations were analyzed within 24 h using a gas chromatograph (GC-2014, Shimadzu, Japan) equipped with appropriate detectors. Flux rates were calculated based on the linear change in gas concentration over the chamber closure period using the Eq. (1). Cumulative emissions were calculated by trapezoidal integration of the flux rates over time using the Eq. (2).

(1) $$F=\rho \frac{V}{A}\frac{T_{0}}{T}\frac{dC_{t}}{d_{t}}$$

where F (µg CH_4_ m^−2^ h^−1^, mg CO_2_ m^−2^ h^−1^ and µg N_2_O m^−2^ h^−1^) represents the emission flux rate of each GHG; ρ (g m^−3^) is the density of each GHG under standard conditions; A (m^2^) is the surface area of the chamber base; V (m^3^) is the volume of the chamber; T_0_ is the absolute temperature under standard conditions; T is the absolute temperature inside the chamber when the gas sample is collected; and (dC_t_/d_t_) represents the rate of change in the concentration of GHG inside the chamber over time (in hours).

(2) $$M=\frac{\left(F_{i+1}+F_{i}\right)}{2}\times \left(t_{i+1}-t_{i}\right)\times \;24\;\times 10^{-2}$$

where M represents the cumulative emission of soil GHG (kg ha^−1^); F_i_ is the soil GHG emissions flux at the (i)-th measurement (µg CH_4_ m^−2^ h^−1^, mg CO_2_ m^−2^ h^−1^, and µg N_2_O m^−2^ h^−1^); and t_i_ is the sampling time at the (i)-th measurement (days).

### 4.3 Soil sampling and analysis

Soil samples were collected at 0–10 cm depth using a soil core sampler (7 cm diameter) from each plot during key growth stages (July and October 2022; January, April, July and October 2023).Each sample was a composite of four random cores. Soil cores were collected adjacent to the chambers. In order not to disturb GHGs emissions, soil in the static chamber were not sampled. Soil samples were transported to the laboratory and processed within 24 h. Each soil sample was equally divided into two subsamples. One subsample was stored at 4 °C for soil microbial and enzyme analyses, while another subsample was used for soil physical and chemistry properties analyses. Soil physicochemical properties were determined using standard methods [9,10]: SOC by the potassium dichromate oxidation method, pH and EC potentiometrically, MBC and MBN by chloroform fumigation-extraction, and enzyme activities by colorimetric methods.

### 4.4 Data analysis

The objective of this data article is to provide the raw dataset to the scientific community for reuse. Accordingly, multivariate statistical analyses (e.g., structural equation modeling, random forest analysis) aimed at inferring causal pathways are beyond its descriptive scope. Such analyses are presented and discussed in the related research article [6].

However, to enhance the utility and interpretability of the dataset, we have performed descriptive statistics (e.g., mean, standard deviation) for key variables across treatments in the dataset file. Furthermore, the dataset is structured in a tidy format with each row representing an observation and each column a variable. This organization, combined with the parallel measurement of greenhouse gas fluxes and soil variables, makes it suitable for future users to conduct analyses the relative contribution and direct/indirect influence paths of soil properties on GHG emissions.

## Limitations

The dataset represents measurements from a single experiment under a specific soil type and climate, which may limit the direct extrapolation of the findings to other regions. While the 1.5-year post-application monitoring provides data on the medium-term dynamics, even longer-term studies (>5 years) are needed to fully capture the fate of biochar and its effects on soil GHG dynamics. Furthermore, the dataset does not include characterization of the soil microbial community structure, which could provide deeper insights into the observed GHG fluxes. It is also important to note that defining an optimal application strategy is a multi-criteria decision that depends on the weighting of goals (e.g., maximizing carbon sequestration, minimizing N_2_O emissions). This dataset does not prescribe a single answer but provides the comprehensive, multivariate evidence base required for such context-specific assessments, including the trade-offs between different outcomes.

## Ethics Statement

The authors confirm that this work does not involve human subjects, animal experiments, or data collected from social media platforms.

## CRediT Author Statement

**Lihong Song:** Conceptualization, Methodology, Validation, Funding acquisition, Writing – review & editing. **Yan Zhang:** Investigation, Formal analysis, Data curation, Writing – original draft.

## Declaration of Competing Interest

The authors declare that they have no known competing financial interests or personal relationships that could have appeared to influence the work reported in this paper.

## Data Availability

FigshareBiochar induced farmland GHGs emission (Original data)
